# Emerging role of Hippo signalling pathway in bladder cancer

**DOI:** 10.1111/jcmm.13293

**Published:** 2017-08-07

**Authors:** Jianling Xia, Ming Zeng, Hua Zhu, Xiangjian Chen, Zhiliang Weng, Shi Li

**Affiliations:** ^1^ Cancer Center Sichuan Academy of Medical Sciences and Sichuan Provincial People's Hospital Hospital of the University of Electronic Science and Technology of China Chengdu Sichuan China; ^2^ Department of Obstetrics and Gynecology The First Affiliated Hospital of Wenzhou Medical University Wenzhou Zhejiang China; ^3^ Department of General Surgery The First Affiliated Hospital of Wenzhou Medical University Wenzhou Zhejiang China; ^4^ Department of Urology The First Affiliated Hospital of Wenzhou Medical University Wenzhou Zhejiang China

**Keywords:** bladder cancer, Hippo pathway, YAP1, TAZ, dysregulation, therapeutic target

## Abstract

Bladder cancer (BC) is one of the most common cancers worldwide with a high progression rate and poor prognosis. The Hippo signalling pathway is a conserved pathway that plays a crucial role in cellular proliferation, differentiation and apoptosis. Furthermore, dysregulation and/or malfunction of the Hippo pathway is common in various human tumours, including BC. In this review, an overview of the Hippo pathway in BC and other cancers is presented. We focus on recent data regarding the Hippo pathway, its network and the regulation of the downstream co‐effectors YAP1/TAZ. The core components of the Hippo pathway, which induce BC stemness acquisition, metastasis and chemoresistance, will be emphasized. Additional research on the Hippo pathway will advance our understanding of the mechanism of BC as well as the development and progression of other cancers and may be exploited therapeutically.

**Table:** 

• **Introduction**
• **The Hippo pathway**
‐ Overview of the Hippo signalling pathway
‐ The network of upstream signals of the Hippo pathway
‐ Regulation of YAP1 and TAZ
Extrinsic regulators
*Cell contact and morphology*
*Extrinsic stress signals*
*G protein‐coupled receptors (GPCRs)*
Intrinsic regulators
*E proteins*
*Cell cycle*
*Other signalling pathways*
• **The Hippo pathway and its role in cancers**
‐ Clinical correlation between the upstream Hippo pathway and human cancers
‐ YAP1/TAZ are key co‐effectors of the Hippo pathway in human cancers
• **Deregulation of the Hippo pathway and its role in bladder cancer**
‐ The Hippo pathway in urinary tract development
‐ The role of the Hippo signalling pathway in bladder cancer
• **Conclusions**
• **Future perspectives**
• **Acknowledgements**
• **Conflict of interests**

## Introduction

BC is the fourth most commonly diagnosed cancer in males. The incidence of BC is about 4 times higher in men than in women 1. More than 70% of patients who have BC are newly diagnosed with non‐muscle‐invasive disease 2. However, after undergoing transurethral resection of the bladder tumour (TURBT) followed by intravesical chemotherapy (22%) or biological therapy with bacillus Calmette‐Guerin (29%) 3, up to 50–70% of cases will experience relapse, and approximately 10–20% will invade into the muscularis propria layer (T2 or greater) 2. For cases with muscle‐invasive disease, treatment options are limited. Cystectomy and chemotherapy combined with radiation are two common options; the long‐term prognosis is poor, however, with a 5‐year survival rate of 47%. For all stages combined, BC patients can expect survival rates of 77% at 5 years and 70% at 10 years 1. High recurrence (range, 50–90%) 4, 5 and progression rates 2 are major obstacles to the treatment of BC. Identifying new therapeutic targets of BC is essential to developing further effective treatment.

The targeting of signalling pathways for cancer treatment has increased in the last decades. However, more therapeutic targets for BC are needed 6. The Hippo signalling pathway, which functions in organ size control, stem cell pluripotency and regeneration 7, has been found to be dysregulated in various human cancers 8, 9, 10, 11, 12, 13. Furthermore, a set of studies have demonstrated the dysregulation of the Hippo pathway in BC 14, 15. This emergence of the Hippo pathway in BC progression may aid in identifying new pharmaceutical targets for BC management. In this review, we first discuss several studies on the roles of the Hippo signalling pathway in embryonic development and human tumours. Then, we detail the various mechanisms of the Hippo pathway in human tumours and in bladder tumours in particular.

## The Hippo pathway

### Overview of the Hippo signalling pathway

**Figure 1:**
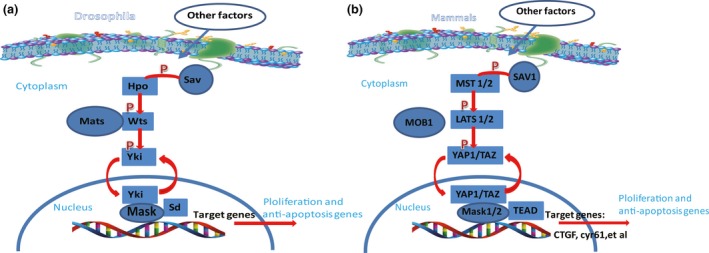
The Hippo signalling pathway in Drosophila and mammals. (**A**) The Drosophila Hippo pathway. In Drosophila, when Yki is relieved from inhibition through phosphorylation‐dependent or phosphorylation‐independent mechanisms, its nuclear translocation then drives target gene expression to regulate cellular proliferation and apoptosis. The phosphorylation mechanism depends on the core kinase cascade including Hpo, Wts, Sav and Mats. (**B**) The mammalian Hippo pathway. In mammals, YAP1 and TAZ localize to the nucleus to interact with TEAD, driving target gene expression to regulate cellular proliferation and apoptosis. After phosphorylation, MST1/2 in turn phosphorylates LATS1/2, facilitated by scaffold proteins SAV1 and MOB1. MOB1 also phosphorylates and activates LATS1/2. Activated LATS1/2 phosphorylate YAP1 and TAZ. YAP1 interacts with Mask1/2 to form complex.

The Hippo signalling pathway, also known as the MST1/2‐WW45‐LATS1/2 signalling pathway, is an important regulator of tissue homeostasis, cell growth and organ size 16, 17. It was initially identified in the fruit fly *Drosophila* in the search for genes essential for cell proliferation, organ growth and decreased apoptosis 18, 19. A specific set of kinases are its key components, including Warts (Wts), Salvador (Sav) and Hippo (Hpo) 18, 20, 21. These genes function as tumour suppressors in *Drosophila*, wherein mutation of these genes leads to dysregulation of cell proliferation and apoptosis (Fig.**** 1) 22. Furthermore, these key components of the signalling pathway are highly conserved in most eukaryotes, from flies to mammals. Deregulation of this pathway, especially mutation of its key components, can activate several oncogenes in cancer cells in various human cancers 8, 9, 10, 11, 12, 23.

### The network of upstream signals of the Hippo pathway

Little is known of the exact mechanisms of the Wts, Sav and Hpo kinases, but cellular polarity may be involved, as these components localize to adherent junctions of polarized epithelial cells 24. Wts mutations lead to dysregulated tissue proliferation 18, 25, whereas Hpo and Sav determine the survival and apoptosis of cells 21, 26, 27. This signalling cascade is conserved from flies to mammals, resulting in a similar network in mammals, including two homologues of Hpo (MST1/2), one homologue of Sav (SAV1), two homologues of Wts (LATS1/2), and two homologues of Yki (YAP1 and its paralog TAZ) 28. Both YAP1 and TAZ are critical transcriptional co‐activators and downstream effectors of the pathway 29, Activation and inactivation of the Hippo pathway depend on activation and inactivation of the kinase cascade. First, RASSF1‐A, a member of the RAS association domain family (RASSF), is involved in the translocation of MST1 to mitochondria. After the stimulation of stress elicits K‐RAS, RASSF1‐A binds to MST1/2 and results in their activation 30, 31. Then, MST1 and MST2 form a complex together with SAV1, facilitating the interaction with LATS1/2 32. This interaction between MST1/2 and LATS1/2 depends on a sequential phosphorylation process. Through suppression of protein phosphatase 2A, MST1/2 are dephosphorylated and stimulate LATS1/2 activation 33, 34, 35, 36. Recently, MOB1 has been shown to play a role in this inactivation 34. In turn, LATS1/2 regulate the interaction between YAP1/TAZ and some important transcriptional target partners, such as the TEA domain‐containing sequence‐specific transcription factors SMAD and RUNX, by regulating the phosphorylation of YAP1 and TAZ. TAZ has been reported to bind to YAP1 37, thereby, exerting their functions on the transcription of various target genes 38, 39. After phosphorylation, YAP1 is retained in the cytosol in a depressed state 38, 40, 41. New research has demonstrated that the multiple ankyrin repeats single KH domain (Mask) is required for the transcriptional output of Yki in *Drosophila* and YAP1 in mammals 42, 43. Mask is conserved in mammals with two homologues, Mask1 (also known as ANKHD1) and Mask2 (also known as ANKRD17)^44^, ^45^.The full activity of Yki or the YAP1/TEAD complex is dependent on the expression of Mask or its mammalian homologue, Mask1. After Mask1 knockdown, YAP1 target genes were substantially suppressed although non‐target genes were not affected 42. (Fig.** ** 1)

### Regulation of YAP1 and TAZ

**Figure 2:**
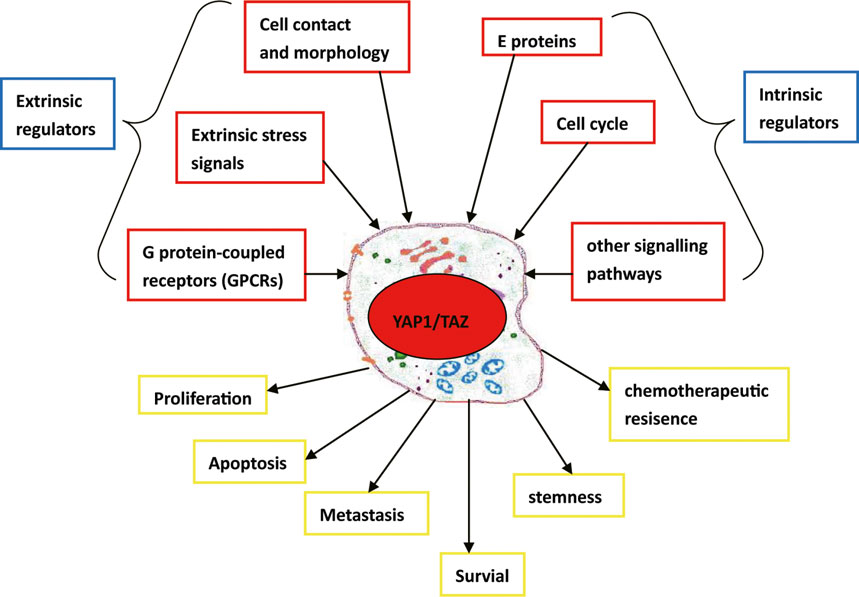
Schematic overview of YAP1/TAZ regulation and function in tumorigenesis.

In addition to the key components of the Hippo pathway, several other intrinsic and extrinsic regulators of YAP1/TAZ have been observed (Fig.**** 2).

#### Extrinsic regulators

##### Cell contact and morphology

Some environmental cues affect YAP1/TAZ activity. For example, in epithelial cells, apical signalling modulates YAP1/TAZ expression through the canonical Crumbs/CRB‐Hippo/MST‐Warts/LATS kinase cascade. When cells differentiate an apical membrane domain, YAP1/TAZ are phosphorylated and inhibited. Although contact occurs between the cells’ extracellular matrixes (ECMs) and basal membrane domains, these two effectors are stimulated 46. The stiffness or elasticity of the ECM has a dramatic effect on F‐actin bundles 47. In *Drosophila* cells, Yorkie activation is positively associated with F‐actin expression 48. In mammalian cells, the maintenance of YAP1/TAZ activity requires a stable role of F‐actin contractility 49. Recent research found that cell morphology can regulate YAP1 nuclear localization. Piezo1, a channel that mediates calcium currents, is crucial for YAP1 nuclear localization through the regulation of cytoskeletal tension 50.

##### Extrinsic stress signals

Considering that the most important role of YAP1/TAZ is to promote cell proliferation and survival 51, a set of extrinsic stress signals, such as endoplasmic reticulum stress, energy stress and hypoxia, has been observed to regulate the Hippo signal pathway. Carbohydrates are the main energy source for cell metabolism. Deran *et al*. have observed that YAP1 and TAZ phosphorylation is rapidly induced by energy stress caused by glucose deprivation 52. Lack of glucose stimulates AMPK activation, which affects the interaction between TEAD and YAP1/TAZ 53. In contrast to oxidative stress, hypoxia seems to affect the interaction between LATS and YAP1/TAZ. Hyperactivity of SIAH2 caused by hypoxia inhibits LATS in a xenograft animal model 54.

##### G protein‐coupled receptors (GPCRs)

Extracellular molecules, such as growth factors and hormones, have been hypothesized to regulate the Hippo signalling pathway so as to control homeostasis. Furthermore, it has been demonstrated that regulation of the Hippo signalling pathway by GPCRs is indeed a common response of cells to hormonal cues 55, 56, 57, 58. GPCRs, along with Rho GTPase and the actin cytoskeleton, can promote or suppress Hippo signalling pathway activity. Whether this results in positive or negative regulation depends on the class of G protein involved. For instance, Gα_12/13_‐ and Gα_q/11_‐coupled GPCRs promote YAP1 and TAZ activity by regulating the actions of Rho‐GTPases, whereas, Gα_s_‐coupled GPCRs inactivate YAP1 by increasing the activities of LATs 28, 59.

#### Intrinsic regulators

Despite these extrinsic regulators, intrinsic transcriptional regulators and signalling pathways are still crucial mediators of the Hippo pathway.

##### E proteins

E proteins are members of the basic helix‐loop‐helix (bHLH) family that mediate cell proliferation, differentiation and commitment in many tissues 60, 61. After binding to the E‐box sequence (CANNTG), E proteins can inhibit ID protein expression. Furthermore, either elevated expression of E proteins or loss of ID proteins can promote Hippo signalling 62. Previous studies have demonstrated that the interaction between E and I proteins can affect various transcriptional factors associated with the Hippo pathway, such as SMAD, TEAD, PAX, HTH and TBX5. These regulators are required for the phosphorylation of YAP1/TAZ and are involved in reducing the activation of the Hippo pathway 39, 63, 64, 65, 66.

##### Cell cycle

LATS1/2 are not only critical components of the Hippo pathway, but they are also regarded as regulators of the cell cycle (G1/S, G2/M and mitosis) 67. Furthermore, the cell cycle can influence the activities of YAP1/TAZ. For instance, during the G2‐M phase, YAP1 and TAZ are phosphorylated at multiple sites by CDK1, which increases cell migration and invasion ability 68.

##### Other signalling pathways

The Hippo signalling pathway is involved in cross‐talk with a number of other signalling pathways, such as the Notch 69, transforming growth factor β (TGF‐β) 70 and Wnt/β‐catenin pathways 71, 72. Among these signalling pathways, most previous studies have focused on the interaction between the Hippo signalling pathway and the Wnt/β‐catenin signalling pathway (Wnt pathway) owing to their obvious roles in tumorigenesis. The Wnt pathway plays critical roles in almost every aspect of embryonic development as well as in homeostasis in various adult tissues. Its germline mutations are associated with a set of human cancers 73. This cross‐interaction was originally reported in 2010 37. In the cytoplasm, both YAP1 and TAZ can directly interact with β‐catenin and suppress β‐catenin nuclear translocation 74, whereas in the nucleus, YAP1 can cooperate with the Wnt pathway to enhance tumorigenicity^75^, ^76^. In the cytoplasm, TAZ interacts with CK1δ/ε and DVL and thereby inhibits the WNT3α‐induced phosphorylation of DVL2. Consequently, the Wnt/β‐catenin signalling pathway is inhibited. Meanwhile, the cytoplasmic accumulation of TAZ can also interact with MST and LATS to inhibit Wnt/β‐catenin pathway‐mediated reporter activity 77. Furthermore, an inhibitory role of the Hippo pathway on the Wnt/β‐catenin pathway in heart development has been demonstrated. Chromatin immunoprecipitation (ChIP) assay results have shown that YAP1‐TEAD and β‐catenin‐TCF/LEF cooperatively regulate some target genes, such as SOX2 and SNAIL2 in heart development 78. In the nucleus, previous studies have demonstrated that the tumorigenicity of deregulated Wnt signalling is dependent on at least two distinct transcriptional complexes: β‐catenin‐YAP1‐TBX5 and β‐catenin‐TCF4 76. Furthermore, the co‐localization of YAP and β‐catenin in the nucleus has been observed in several colorectal cancer cell lines 75.

## The Hippo pathway and its role in cancers

### Clinical correlation between the upstream Hippo pathway and human cancers

Numerous retrospective analyses of tumour specimens have demonstrated a significant association between aberrant expression of Hippo pathway components and cancer clinical stages (**Table** 1). For instance, MST1/2 and LATS1/2, the upstream kinases of the Hippo pathway, function as tumour suppressors in multiple human cancers 79. In three different murine syngeneic tumour models (B16, SCC7 and 4T1), knockout of LATS1/2 in tumour cells inhibits proliferation. Mechanistically, LATS1/2‐null tumour cells secrete nucleic acid‐rich extracellular vesicles, which induce a type I interferon response *via* the Toll‐like receptor MYD88/TRIF pathway, thus improving tumour immunogenicity 80. MST1/2 expression is explicitly correlated with increased clinical stage in gastrointestinal cancers 81, 82, 83, 84, 85. Mask1/2 play a crucial role in various human cancers. Mask1 is critical in prostate cancer, myeloma and leukaemia 86, 87, 88, whereas elevated Mask2 expression has been observed in BC 89.

**Table 1:** Dysregulated Hippo pathway components in human tumours

Hippo pathway component	Cancer type	Role in human tumours	Reference
MST1/2	Gastric cancer	Invasion, metastasis, higher clinical stage, and poorer prognosis	10, 79, 80, 81, 82, 83, 84, 85, 133
	Colorectal cancer		
	Hepatocellular cancer		
	Breast cancer		
	Gastric cancer		
LATS1/2	Prostate cancer	Proliferation, metastasis, increased clinical stage, reduced overall survival, and recurrence‐free survival	79, 80, 82, 84, 130, 133
	Renal cancer		
	Non‐small lung cancer		
	Colorectal cancer		
	Gastric cancer		
	Bladder cancer		
Mask1/2	Prostate cancer	Proliferation, migration	86, 87, 88, 89
	Myeloma		
	Leukaemia		
	Bladder cancer		
YAP1	Bladder cancer	Proliferation, invasion, metastasis, higher clinical stage, reduced overall survival, metastasis‐free survival, and chemotherapy resistance	12, 14, 15, 89, 134, 136
	Gastric cancer		8, 84, 124, 132
	Colorectal cancer		75, 82, 125, 126
	Squamous cell carcinoma		116
	Non‐small cell lung cancer		9, 92, 119
	Ovarian cancer		11, 85
	Uveal melanoma		93
	Endometrial cancer		94
	Hepatocellular cancer		69, 95, 107, 109, 110
	Pancreatic ductal adenocarcinoma		68, 96
	Cholangiocarcinoma		91
	Head and neck cancer		90
	Breast cancer		58, 115
	Malignant mesothelioma		70
	Prostate cancer		86
	Endometrial cancer		94
	Medulloblastomas		114
	Meningiomas		139
TAZ	Hepatocellular cancer	Proliferation, invasion, metastasis, higher clinical stage, shorter overall survival, disease recurrence, poor prognosis and chemotherapy resistance	97, 107
	Retinoblastoma		102
	Gastric cancer		84, 123
	Colon cancer		82, 101
	Oral cancer		98
	Ovarian cancer		104
	Endometrial cancer		94, 103
	Osteosarcoma		105
	Non‐small cell lung cancer		100, 117, 119
	Breast cancer		40, 58, 111, 138
	Tongue squamous cell carcinoma		99
	lioma		106
	Bladder cancer		136

### YAP1/TAZ are key co‐effectors of the Hippo pathway in human cancers

As the most important effectors, YAP1 and its closely related paralog TAZ act as oncogenes in various human cancers. Up‐regulation of YAP1 has been observed in gastric cancer, colorectal cancer, squamous cell carcinoma (SCC), non‐small cell lung cancer (NSCLC), ovarian cancer, uveal melanoma, endometrial cancer, hepatocellular cancer (HCC), pancreatic ductal adenocarcinoma, cholangiocarcinoma, and head and neck cancer 9, 82, 84, 90, 91, 92, 93, 94, 95, 96. Meanwhile, overexpression of TAZ is observed in HCC, retinoblastoma, gastric cancer, colon cancer, NSCLC, ovarian cancer, endometrial cancer, osteosarcoma, glioma and oral cancer 84, 94, 97, 98, 99, 100, 101, 102, 103, 104, 105, 106.

In liver cancer, several studies have demonstrated that elevated expression of YAP1/TAZ is associated with higher pathological grades and poor clinical differentiation 97, 107. In transgenic mice, YAP1 overexpression results in hepatomegaly and liver cancers similar to human HCC 108. By interacting with β‐catenin, hydrodynamic transfection of YAP1 promotes liver tumour development 109. In line with this, up‐regulation of YAP1/TAZ in a normal human liver cell line, MHIA, endows tumorigenic properties 110.

Among a database of breast cancer patients, a significant correlation was observed between high pathological grade, metastatic proclivity, carcinoma stemness and poor outcome 111, 112, 113. Breast cancer originates in the epithelial cells of the mammary gland, and YAP1 can promote epithelial‐mesenchymal transition (EMT) and proliferation in breast cancer cell lines 114. In a mouse model, YAP1/TAZ cooperate with Her2, Polyoma‐middle T and Wnt1 to induce breast cancer development 115.

In NSCLC, significant correlations have been demonstrated between up‐regulation of YAP1/TAZ and malignant features (high histological grade, late TNM stage and poor prognosis) 116, 117. By binding with OCT4 through its WW domain, YAP1 promotes SOX2 activity and thus leads to maintenance of tumour stemness 118. Furthermore, after knockdown of the oncogene KRAS^G12D^, non‐metastatic tumours in LAC mice display weaker YAP1/TAZ staining compared with that in metastatic samples 119, 120.

In gastric cancer, deregulation of the Hippo signalling pathway is significantly correlated with initiation, development and distant metastasis of gastric cancer^84^. Elevated expression of *YAP1* mRNA and YAP1 protein levels both in the nucleus and the cytoplasm was originally observed in high‐grade or metastatic gastric cancer samples 121. The up‐regulation of YAP1 can promote RAF/MEK/ERK pathway activities and thus enhance the expression of c‐FOS in gastric cancer cells 8. Furthermore, RUNX2, a Runt box domain DNA‐binding transcription factor, interacts with YAP1 to inhibit p21 expression, increasing oncogenic properties 122. Similarly, high expression of TAZ has been observed in human gastric cancer 123. Following the disruption of the interaction between TAZ and TEADs, the proliferation of gastric cells is inhibited both *in vivo* and *in vitro* 124.

Elevated expression of YAP1 has been observed among cases in four databases of colorectal cancer patients 125. In line with this finding, associations between YAP1/TAZ overexpression and poor prognosis and drug resistance have also been reported 126. Deregulation of the Wnt/β‐catenin signalling pathway is commonly observed in colorectal cancer, which is significantly correlated with the Hippo pathway 127. Among 36 colorectal cancer specimens, up to 86% scored positively for YAP1 and β‐catenin expression 75. In HCT 116 and advanced colorectal cell lines, activation of Wnt/β‐catenin is dependent on endonuclear YAP1 expression 75.

Above all, the key co‐effectors YAP1/TAZ are responsible for various key attributes of many different human cancers. YAP1/TAZ function in tumour cell proliferation, survival, metastasis and stemness (Fig.**** 2).

## Deregulation of the Hippo pathway and its role in bladder cancer

### The Hippo pathway in urinary tract development

The most important step in urinary tract development is the movement of the ureter from its initial branch point on the nephric duct (ND) to its final insertion site in the cloaca (primitive bladder and urethra) 128. Proteins in the Hippo signalling pathway, especially YAP1 and TAZ, play an essential role in urinary tract development. After silencing of YAP1 in the ND, most newborn mice die within 24 hrs owing to bladder absence or kidney anomalies 129. Furthermore, YAP1 is essential in the progress of the ureter from ND insertion to the bladder and the development of bladder. YAP1 deletion also results in an abnormal junction between the ureter and bladder 129. These studies highlight the crucial role of the Hippo pathway in urinary tract development.

### The role of the Hippo signalling pathway in bladder cancer

Deregulation of the Hippo pathway is significantly correlated with the initiation, development and metastasis of BC (**Table** 2) 14. MST1/2 and LATS1, the most upstream proteins in the Hippo signalling pathway, act as tumour suppressors of human cancers. Down‐regulation of LATS1 and MST1/2 has been demonstrated in human BC 130, 131. *LATS1* mRNA levels were remarkably low in 12 urinary BC specimens from Egyptian patients^130^. Another tumour suppressor, Runt‐related transcription factor 3 (RUNX3), is also an conserved component of this signalling pathway 131, 132. The interactions among RUNX3, MST1/2 and SAV1 are very complicated. SAV1 initially promotes the interaction between RUNX3 and MST2. In turn, MST2 re‐enhances the activation of SAV1 and RUNX3. Finally, activation of these three components inhibits cell proliferation. After *RUNX3* knockdown using siRNA, MST1/2‐mediated cell death was abolished 131, 133. The TEAD‐YAP1 complex is crucial for YAP1 function in various cancers. Research has demonstrated that RUNX3 abrogates the ability of TEAD to bind DNA and thus deregulates TEAD‐YAP activity 132. Recently, a novel cofactor of the TEAD‐YAP complex, named Mask1/2, was identified. Elevated YAP1 expression is able to enhance expression of the target genes (*CTGF*,* cyr61*,*et al*.) and promote BC cell growth and migration, whereas Mask2 knockdown suppresses these genes 89.

**Table 2:** Summary of clinical correlations between dysregulated Hippo pathway components and bladder cancer

Hippo pathway component	Role in bladder cancer development	References
MST1/2	MST1/2 and RUNX3 collaborate and mediate BC cell death	132, 133, 134
RUNX3	Complicated interaction among MST1/2, RUNX3 and SAV1 deregulate the YAP‐TEAD activity and is crucial in BC cell proliferation and apoptosis	
LATS1	Remarkably low level in BC tissues	131
	Alterations of single base pairs in this gene are observed	
Mask2	Mask 2 is required for YAP‐induced BC cell growth and migration	89
YAP1	Elevated YAP1 expression significantly associates with poor clinicopathologic stage and adverse patient survival	14, 15
	Further, YAP1 expression is inversely correlated with chemotherapy sensitivity	142
TAZ	TAZ together with YAP1 protect KLF5 from degradation in BC	137
	Knockdown of KLF5 induces BC cell apoptosis	

As the key downstream effector, YAP1 and its paralog TAZ also play crucial roles in human BC. *YAP1* mRNA and YAP1 protein levels were first observed to be dramatically up‐regulated in urothelial carcinoma of the bladder, especially in high‐grade and metastatic samples 14. Furthermore, this study also provided evidence that YAP1 can act as an biomarker for BC because of the significant correlation between elevated YAP1 expression and adverse patient survival. Interestingly, another study observed that nuclear YAP1 and cytoplasmic pYAP1 levels are lower in BC tissues compared to those of normal urothelial tissues 134. I have also explored the mechanism of YAP1 in BC 15. In my opinion, total YAP1 expression is up‐regulated in bladder tumours. After being phosphorylated by LATS, pYAP1 remains in the cytoplasm. Only unphosphorylated YAP1 translocates into the nucleus and functions as an oncogene 135. This can explain differences in the expression of YAP1 and pYAP1 in the cytoplasms and nuclei of carcinoma cells. The co‐partner of YAP1, TAZ, is also activated in BC. In BC, KLF5 acts as an oncogene that promotes cell proliferation; YAP1/TAZ are capable of preventing KLF5 protein degradation 136.

Previous studies have demonstrated that YAP1/TAZ play crucial roles in cancer stem cells 137. Indeed, carcinoma cells with activated YAP1/TAZ are resistant to chemotherapeutic drugs. In a set of human tumours including breast cancer, meningiomas and lung cancer, YAP1/TAZ are capable of maintaining cancer cell stemness and protecting carcinoma cells from chemotherapeutic drugs 116, 138, 139, 140. In BC, platinum‐based chemotherapy is required for treatment of muscle‐invasive BC patients in the perioperative period. In urothelial carcinoma patient‐derived xenograft models, YAP1 expression is inversely correlated with cisplatin sensitivity. Furthermore, *in vitro* experiments found that DNA damage is not efficiently repaired in YAP1 knock‐down cells. Furthermore, in YAP1‐silenced cells, a significant increase in cell death was observed after cisplatin treatment 141. A more thorough understanding of the mechanisms leading to YAP1 activation during the acquisition of drug resistance would be helpful in developing new treatment strategies.

Furthermore, a set of essential oncogenes in BC can be regulated by the Hippo pathway. *P53* 142 and *c‐Myc* 143 are significantly associated with BC progression. YAP1 is also a cofactor of *p73*, a member of the *p53* tumour suppressor family 144; upon DNA damage, *p73* interacts with YAP1 through its PPPY motif 145. A recent study revealed a unique positive auto‐regulatory feedback loop underlying the interaction between YAP1 and *c‐Myc* in liver cancer 146. EMT has been identified as a crucial event in the pathogenesis of BC 147 and is also mediated by YAP1 148. Recently, the long non‐coding RNA H19 (lncRNA H19) has been regarded as an important biomarker in BC 149. We have previously explored the correlation between YAP1and H19 in BC 15.

## Conclusions

The Hippo signalling pathway is an evolutionarily conserved regulator of cell proliferation, apoptosis, organ growth and tissue homeostasis. The function of the Hippo signalling pathway is regulated by a set of intrinsic and extrinsic regulators and also involves cross‐talk with multiple other signalling pathways. Most components of the Hippo pathway, especially the key downstream effectors YAP1/TAZ, act as crucial regulators in various human cancers. In BC, deregulation of the Hippo signalling pathway is correlated with clinicopathological characteristics and prognoses. The Hippo signalling pathway has an essential effect on the proliferation, metastasis and drug resistance of BC. We therefore suggest that the Hippo signalling pathway could be a potential source of functional biomarkers and new therapeutic targets in BC, as well as in many other cancers.

## Future perspectives

Much more research needs to be done on various aspects of BC. First, the Hippo signalling pathway, which has already received much attention, demands greater investigation in the field of oncology. Furthermore, the upstream components of the Hippo signalling pathway (other than YAP1/TAZ) in particular require further research and may be important in tumour development. Second, in addition to its effects on cell proliferation, metastasis and chemotherapeutic drug resistance, the effects of the Hippo pathway on lymphangiogenesis, autophagy, angiogenesis and the Warburg effect in BC cells should also be defined. Third, the detailed mechanisms or other factors that are involved in BC processes should be explored further, even though some oncogenes and signalling pathways have already been confirmed to cooperate with the Hippo signalling pathway in BC progression, as multiple intrinsic and extrinsic regulators can affect the activities of the Hippo pathway. It is not clear why so many different factors join to activate the same signalling pathway. A possible explanation is that there are distinct molecular gatekeepers that must be bypassed. Some factors ensure the activity of entry‐level pathway effectors, whereas others inhibit their functions. Thus, different combinations of regulators participate in regulating the signalling pathway in cancer progression. Furthermore, under some specific conditions, YAP1 may switch from an oncogene to an anti‐oncogene. The mechanisms of YAP1 and phosphorylated YAP1 activity in the cytoplasm and nucleus require further investigation. Finally, and most importantly, the Hippo signalling pathway is involved in the development of chemotherapeutic resistance. Specific blockers or antagonists that specifically act on certain components of the Hippo signalling pathway with few side effects should be developed to translate basic research findings into clinical applications. The currently available agents that primarily act on the Hippo pathway are not fully satisfactory as some have limited effects and some are not specific, causing various adverse clinical effects. The development of new drugs that act on the Hippo signalling pathway is urgently needed, although large‐scale studies should be developed before clinical applications are implemented in BC patients.

Above all, the use of proteins the Hippo signalling pathway as diagnostic, prognostic or therapeutic targets for BC is recommended. Although some progress has been achieved in this area, more work remains to be carried out, especially regarding the development of new agents for BC treatment.

## Conflict of interests

The authors confirm that there are no conflicts of interest.
